# Use of a Diagonal Approach to Health System Strengthening and Measles Elimination after a Large Nationwide Outbreak in Mongolia

**DOI:** 10.3201/eid2313.170594

**Published:** 2017-12

**Authors:** José E. Hagan, Ashley Greiner, Ulzii-Orshikh Luvsansharav, Jason Lake, Christopher Lee, Roberta Pastore, Yoshihiro Takashima, Amarzaya Sarankhuu, Sodbayar Demberelsuren, Rachel Smith, Benjamin Park, James L. Goodson

**Affiliations:** Centers for Disease Control and Prevention, Atlanta, Georgia, USA (J.E. Hagan, A. Greiner, U.-O. Luvsansharav, J. Lake, C. Lee, R. Smith, B. Park, J.L. Goodson);; World Health Organization Regional Office for the Western Pacific, Manila, the Philippines (R. Pastore, Y. Takashima);; Ministry of Health and Sports, Ulaanbaatar (A. Sarankhuu);; World Health Organization Mongolia Country Office, Ulaanbaatar (S. Demberelsuren)

**Keywords:** immunization, measles, outbreak response, elimination, measles virus, viruses, measles elimination, health systems, Mongolia, global health security, prevent-detect-respond

## Abstract

Measles is a highly transmissible infectious disease that causes serious illness and death worldwide. Efforts to eliminate measles through achieving high immunization coverage, well-performing surveillance systems, and rapid and effective outbreak response mechanisms while strategically engaging and strengthening health systems have been termed a diagonal approach. In March 2015, a large nationwide measles epidemic occurred in Mongolia, 1 year after verification of measles elimination in this country. A multidisciplinary team conducted an outbreak investigation that included a broad health system assessment, organized around the Global Health Security Agenda framework of Prevent-Detect-Respond, to provide recommendations for evidence-based interventions to interrupt the epidemic and strengthen the overall health system to prevent future outbreaks of measles and other epidemic-prone infectious threats. This investigation demonstrated the value of evaluating elements of the broader health system in investigating measles outbreaks and the need for using a diagonal approach to achieving sustainable measles elimination.

Measles, a highly transmissible infectious disease that causes serious illness and death worldwide, is often referred to as a public health “canary in the coalmine” because it can be used as both a signal of weak health systems and a driver for strategies and policies to strengthen health systems (*1*). When programmatic weaknesses in immunization systems occur, measles is frequently the first vaccine-preventable disease (VPD) detected (*2*–*5*). Moreover, because of the high transmissibility of measles virus, the recognizable clinical presentation of nearly all cases in high-incidence settings, the high efficacy of the vaccine for prevention, and lifelong immunity after vaccination or acute infection, measles epidemiology generally reflects population susceptibility and indicates vulnerable communities, areas with lack of response capacity, and weaknesses in the health system (*6*,*7*). Measles elimination, therefore, becomes a useful vehicle to achieve broad strengthening of the overall health system (*8*). The “canary in the coalmine” approach to measles elimination efforts takes advantage of vertical strategies that focus on using surveillance data for action and to identify areas missed by vaccination, and of horizontal strategies that build systems and health services to sustain the gains and achieve broader objectives. The combination of these approaches has been described as a diagonal approach (*9*).

The Global Vaccine Action Plan (GVAP), approved by the World Health Assembly in 2012, set targets for vaccination coverage and a goal to achieve measles and rubella elimination in 5 of the 6 World Health Organization (WHO) regions by 2020 (*10*). In 2012, the Measles & Rubella Initiative partners launched the Global Measles and Rubella Strategic Plan 2012–2020 with targets aligned to the GVAP (*11*). Measles-driven policies and elimination strategies can provide opportunities for improving immunization service delivery performance, as well as strengthening health systems to help achieve the United Nations Sustainable Development Goals and Universal Health Coverage (*8*,*9*,*12*). The Global Health Security Agenda (GHSA) is a partnership between governments, multilateral organizations, and civil society launched in 2014 to promote global health security against infectious disease threats and drive full implementation of the WHO International Health Regulations (IHR 2005) (*13*), organized within a framework of Prevent-Detect-Respond (*14*). Recognizing that immunization is a key requirement to advancing global health security (*12*), the framework includes monitoring of measles vaccination coverage as a GHSA performance indicator, dovetailing with ongoing efforts to increase vaccination coverage and achieve measles elimination (*11*,*15*).

Mongolia, a WHO member state in the Western Pacific Region (WPR), participates in the GHSA (*16*) and has received support to strengthen IHR 2005 capabilities and response capacity for public health events of international concern. In 2009, Mongolia established an Early Warning, Alert, and Response Network (EWARN) (*17*) to supplement existing disease-specific, case-based surveillance systems by collecting syndromic event-based data from public primary health facilities. With GHSA support, a national public health Emergency Operations Center (EOC) and corresponding Incident Management System (IMS) were established in 2015 to coordinate response activities, particularly during outbreaks. In addition, satellite emergency response hubs termed Emergency Operations Points (EOPs) were established at national public health agencies.

In March 2014, the WHO WPR Verification Commission for Measles Elimination verified that measles elimination, which is defined as no measles case reported for 36 months in a country meeting required program performance indicators (*18*), had been achieved in Mongolia. However, in March 2015, multiple laboratory-confirmed measles cases were detected in the capital city, Ulaanbaatar; by June 5, 2015, a total of 11,181 suspected cases had been reported nationwide from all 21 provinces (*19*). The government of Mongolia requested that WHO and the US Centers for Disease Control and Prevention, in collaboration with the Ministry of Health and Sports (MOHS), conduct an outbreak investigation to assess factors contributing to ongoing transmission and provide recommendations for outbreak response and elimination strategies. In addition to identifying risk factors for transmission and evaluating the response vaccination activities and strategies, we used the measles outbreak as an opportunity to conduct a broader evaluation of the health system and emergency response strategies, following the GHSA framework, to prevent future outbreaks in Mongolia. Because nosocomial transmission of measles virus was identified early in the investigation as being a possible contributor to the outbreak, we conducted an assessment of infection prevention and control (IPC) practices in select healthcare facilities (HCFs). We also reviewed surveillance data, standard operating procedures (SOPs), and practices, and evaluated national emergency preparedness activities and response processes during the outbreak.

## Methods

### Outbreak Investigation

To better describe the epidemiology of healthcare-associated measles and to identify and recommend prevention measures, we reviewed data from case-based surveillance for March 1, 2015–June 26, 2016. Confirmed cases were either laboratory confirmed by positive test result for measles-specific IgM ELISA or PCR or clinically confirmed by meeting criteria of rash plus fever and >1 of the following: cough, coryza, or conjunctivitis. We also reviewed National Center for Communicable Diseases (NCCD) measles surveillance data for cases with onset during December 1, 2015–June 27, 2016, during which period-specific healthcare exposures were collected for case-patients. We defined healthcare-associated cases as laboratory-confirmed measles virus infection in a patient who was a healthcare worker (HCW) or who was hospitalized (non-HCW) during the 7–21 days (measles incubation period) preceding onset of signs or symptoms and who had an epidemiologic link to a hospitalized case-patient or lacked a known community source.

### Assessment of IPC Policies and Practices (Prevent)

We assessed IPC practices at 3 hospitals in Ulaanbaatar with a large number of reported outbreak cases in surveillance data: 2 national referral tertiary care hospitals (1 of which was NCCD, the national HCF for infectious diseases) and 1 district hospital. We also assessed 1 primary care facility. At the 4 selected HCFs, we conducted structured interviews of facility staff and directly observed IPC practices and compliance with MOHS guidance and recommendations from previously published IPC documents (*20*–*25*). We reviewed MOHS occupational health policy, MOHS bulletins to HCFs, and HCF occupational health policies to evaluate vaccination and furlough policies.

### Assessment of Surveillance (Detect)

We reviewed policies, SOPs, and protocols, conducted key informant interviews, and analyzed data for January 1, 2014–June 27, 2016. We used this information to assess national laboratory-supported measles case-based surveillance and EWARN surveillance for fever and rash syndrome.

### Assessment of Emergency Preparedness and Outbreak Response (Respond)

We conducted interviews with key stakeholders at national and subnational levels of the emergency response system and reviewed EOC, EOP, and IMS SOPs. We identified and mapped roles, responsibilities, and mechanisms and verified them with stakeholders. After the investigation, we held a consultative training workshop with MOHS, NCCD, and Mongolia Field Epidemiology Training Program (FETP) staff to formulate specific recommendations on the basis of evidence from the investigation findings.

## Findings and Recommendations

### Outbreak Investigation

**Figure 1:**
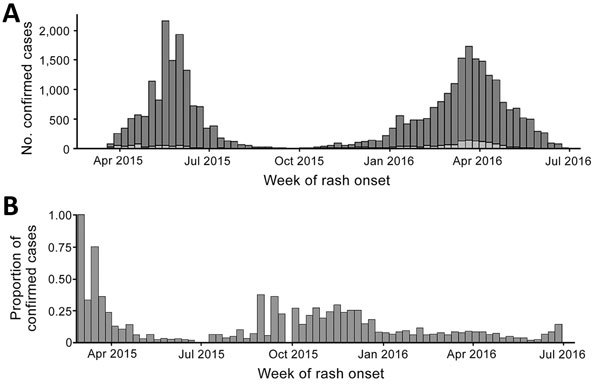
Confirmed measles cases in Mongolia, March 1, 2015–Jun 27, 2016. A) Confirmed cases by epidemiologic week of rash onset and reported exposure to a healthcare facility during the 7–21 days (measles incubation period) before rash onset. B) Proportion of confirmed case-patients by epidemiologic week of rash onset and reported exposure to a healthcare facility during the measles incubation period. Light gray indicates healthcare exposure during incubation period; dark gray indicates no exposure or unknown. Cases were confirmed by laboratory results (positive IgM ELISA or PCR) or clinical criteria (rash plus fever and >1 of the following: cough, coryza, or conjunctivitis).

Of 33,947 confirmed case-patients with rash onset during March 1, 2015–June 27, 2016, a total of 14,407 (42%) were hospitalized and 2,222 (7%) reported visiting an HCF during the incubation period before rash onset, particularly during the initial phase of each of the 2 waves of intense transmission in 2015 and 2016, when ≈25% of cases had HCF exposure (Figure 1). During December 1, 2015–June 27, 2016, we identified 603 total healthcare-associated measles cases. Of these, 55 (9%) occurred in HCWs; 220 (36%) occurred in infants >9 months of age who were eligible for routine measles vaccination; and 448 (74%) occurred in infants >6 months of age who were therefore eligible for postexposure or outbreak response measles vaccination.

### Prevent: IPC Assessment

Some IPC policies were available, but lack of corresponding infrastructure limited proper infection control to prevent measles virus transmission in hospitals. For example, we found inconsistent implementation of appropriate procedures for isolation or cohorting of confirmed measles cases; in addition, no negative pressure isolation rooms existed in any of the HCFs visited, and only 1 airborne isolation room existed in the country.

Policies and SOPs for measles contact tracing and postexposure prophylaxis (PEP) in HCFs existed; however, these recommendations were generally not practiced during the outbreak. The National Standard on Measles Surveillance Guidelines from 2003 recommended routine contact tracing of measles cases and, where appropriate, administration of measles-containing vaccine (MCV) or immunoglobulin as PEP (*26*). However, we found that contact tracing efforts in HCFs became quickly overwhelmed by the increasing case counts, primarily because of limited financial and human resources. Specific guidance for measles PEP in HCFs was not provided during the outbreak, and MCV and immunoglobulin supplies were not made available for PEP.

Occupational health safeguards to prevent measles generally were not present. Proof of measles vaccination was not a mandatory condition of employment in HCFs; records of measles immunity status were not routinely kept at HCFs. Although MCV was reportedly offered to HCWs during the outbreak, we found inconsistent provision of the vaccine for HCWs, and records of staff vaccination during the outbreak were not available to review. Nonimmune HCWs were not furloughed or temporarily reassigned from patient care activities after measles exposure, unless and until febrile rash illness developed. In addition, HCWs who were furloughed did not receive a salary during the furlough period. Therefore, HCWs likely worked providing care to patients during the highly contagious period that begins 4 days before rash onset and lasts until 4 days after rash onset.

### Detect: Disease Surveillance Assessment

According to surveillance protocols, cases detected by EWARN meeting the syndromic case definition of fever with maculopapular rash are investigated and also reported through the measles case-based surveillance system, using an individual case investigation form and collecting a specimen for laboratory testing for case confirmation. Surveillance protocols did not distinguish between appropriate procedures for routine surveillance and enhancements to surveillance that are needed during outbreaks and did not include parameters on when to scale back specimen collection or how to perform epidemiologic linkage for case confirmation.

Cases from epidemiologic and laboratory surveillance databases were not linked by using the standard practice of assigning unique identifiers to each case and specimen. More than 14,000 specimens were collected and tested during this outbreak, overwhelming the national reference laboratory and leading to delays in case confirmation. Epidemiologic linkage was not performed uniformly or according to the WHO WPR recommended case classification algorithm (*27*). Trends in EWARN and case-based surveillance were not routinely compared, and compatible cases detected by EWARN were not consistently reported and investigated through the case-based system. EWARN data indicated an initial increase in fever and rash cases beginning in epidemiologic week 17 of 2014. However, we found discrepancies between EWARN and case-based data in 2014, with much lower sensitivity in the case-based system, possibly leading to delayed detection of initial cases as many suspected cases were not investigated and tested. The first confirmed cases were detected in epidemiologic week 9 of 2015, in Ulaanbaatar and in Umnogovi Province, bordering China.

### Respond: Emergency Preparedness and Outbreak Response Assessment

The IMS SOPs and staffing needs for the national EOC and HCF EOPs were still under development at the time of the outbreak, which limited the coordination capacity of the IMS during the outbreak. The EOC was not staffed until May 2016, as the outbreak was winding down, and even once staffed, it was never activated. Relationships between and roles of the EOC and EOPs were not clearly delineated. There was limited preallocation of resources and funding to the EOC and EOPs in the event of a public health emergency, delaying and constraining response activities. The response lead, termed the Event Manager in Mongolia, did not have the authority to release funds or resources without substantial review by supervisors, also delaying response activities. Frequent reassessments and/or risk assessments of the outbreak and response activities to ensure that needs matched the available resources were not performed. Finally, no national outbreak preparedness and response plan existed that identified the basic needs for measles outbreaks (i.e., vaccination, airborne precautions, laboratory support) or SOPs outlining airborne disease outbreak response activities.

**Figure 2:**
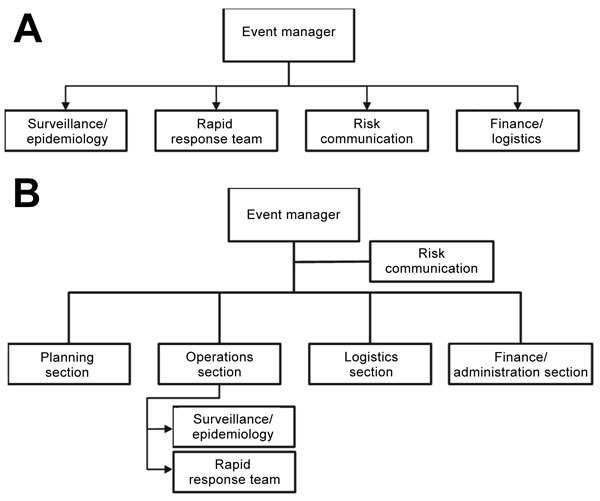
Flowcharts for organization of the Incident Management System in Mongolia during (A) and after (B) the 2015–2016 measles outbreak. Restructuring of the system after the outbreak was designed to better align with World Health Organization recommendations (*28*). Note that this figure does not represent a complete Incident Management System, only a restructuring of the existing system.

The NCCD EOP was formally activated in December 2015 to lead the measles outbreak response. The NCCD EOP used a draft IMS proposal, and although the draft covered basic sections required in a public health emergency response (logistics and finance sections), the structure (Figure 2, panel A) did not mirror standard IMS structure as recommended by WHO (*28*). In addition, critical organizational subdivisions required for a successful measles outbreak response were not delineated in the structure, such as the inclusion of operations teams to support epidemiologic investigation (case investigation and contact tracing) and IPC activities (Figure 2, panel B).

Response demands exceeded the capacity of available NCCD EOP staff and resources, especially at the outbreak peak. No staff roster or surge capacity were available to mobilize staff from other national agencies that had applicable skill sets (e.g., epidemiologists, intensivists, logisticians, laboratorians, FETP) to address this deficit.

### Selected Recommendations

As a result of our investigation, we developed several recommendations. These recommendations addressed the gaps in policy, practice, and infrastructure identified as likely contributing causes of the outbreak and sustained virus transmission.

#### Prevent

Recommendations for long-term systems strengthening included improving physical building infrastructure necessary for proper IPC of measles and other contagious respiratory diseases. In the short term, measles contact tracing and PEP in HCFs should be implemented according to existing national guidelines. MCV and immunoglobulin for PEP should be stockpiled and mechanisms developed for rapid mobilization and delivery once a measles outbreak is confirmed. To limit healthcare-acquired transmission during measles outbreaks, only staff who have 2 documented MCV doses or evidence of immunity through serologic testing should be allowed to interact with patients (*29*). HCFs should maintain records of staff measles immunity status, proactively identify staff without immunity, and provide MCV. HCWs should be encouraged to remain at home when they feel ill and should not suffer financial losses for doing so.

#### Detect

A comprehensive surveillance review should be conducted to identify gaps in surveillance performance and improve data flow to decision makers for prompt, effective action. One of our key recommendations was to establish coordination mechanisms to align EWARN and case-based VPD surveillance systems so that cases are adequately and promptly investigated and so that trends in one surveillance system trigger enhanced surveillance mechanisms in the other surveillance system. In addition, we recommended improved linkage between epidemiologic investigation and laboratory testing, appropriate use of a unique identifier variable, and case classification including epidemiologic linkage for case confirmation.

#### Respond

We recommended that a national outbreak preparedness and response plan for measles and other airborne infectious diseases be established and agreed upon by all relevant stakeholders. Emergency response SOPs should be finalized as an urgent preparedness activity to map out the organizational structure per WHO recommendations, define the interaction of the EOC and EOPs, delineate procedures for activation and deactivation, define roles and responsibilities of positions in the IMS structure, and outline data flow and communication mechanisms with national and subnational staff and partner agencies (*28*). Emergency response SOPs should incorporate lessons learned from previous outbreaks and should be distributed to all stakeholders at each level of the public health system, including primary health clinics. The IMS could be strengthened by mapping out further subdivisions that are required for an effective outbreak response for airborne diseases (Figure 2, panel B). The EOC and EOPs should implement training for staff members regarding their specific roles in emergency response and run periodic exercises, using a mock measles outbreak scenario, to test the SOPs and emergency response capacity and coordination with relevant public health stakeholders outside of the EOC and EOPs. Systems breakdowns identified through these activities should lead to the refinement of emergency preparedness and response guidelines and other relevant SOPs, such as those for IPC and surveillance.

## Conclusions

Until measles is eradicated worldwide, the risk for measles virus importations and subsequent outbreaks will remain in countries such as Mongolia that have achieved measles elimination. Prevention of large measles outbreaks that may occur after virus importations can be achieved by implementing measles elimination strategies, maintaining high 2-dose measles vaccination coverage, and developing robust capacity for rapid response. Measles outbreaks in postelimination settings can provide valuable lessons on how to prevent and overcome hurdles on the road to eradication and can reveal weaknesses in health systems that might undermine control efforts for other infectious diseases.

Preventing and controlling measles outbreaks require established policies and procedures that pay special attention to specific settings where measles virus introduction and sustained transmission may occur. For example, HCFs can serve as amplification points for outbreaks of measles and other infectious diseases (*30*–*33*). Given the universal challenges of efforts to quickly identify cases of infectious diseases and appropriately triage patients in busy HCFs, vaccination of all HCWs and use of PEP should be prioritized because these methods are likely the most effective strategies to prevent and reduce healthcare-associated measles.

Rapid detection and response to measles outbreaks is essential for elimination efforts and can prevent infections and reduce the number of deaths (*34*,*35*). The IHR 2005 and GHSA frameworks outline guidance on surveillance system strengthening to ensure countries have the capacity to detect and respond to outbreaks of VPDs such as measles, as well as new and emerging pathogens (*13*,*36*). As a part of this guidance, syndromic surveillance systems such as EWARN should be used to provide sensitive signals of major public health events but must be linked to systems for immediate case investigation, confirmation, and coordinated response activities, ideally through an incident management system or its equivalent. EWARN should be tightly linked with case-based surveillance through routine data sharing. Case-based surveillance is a key requirement for achieving measles and rubella elimination and provides the added benefit of being a standard against which signals from parallel syndromic surveillance systems such as EWARN can be checked and calibrated.

Achieving successful control of measles outbreaks requires a multifaceted strategy involving surveillance, laboratory capacity, contact tracing, vaccination, and hospital IPC measures; thus, maintaining a capacity for coordination of activities is critical for an effective, cohesive outbreak response (*37*). During measles outbreaks, the speed and completeness of response measures are critical and dictate the extent of measles transmission and burden of disease (*38*). Delaying or poorly implementing response efforts such as contact tracing and targeted vaccination can lead to an exponential increase in additional exposures, infectious cases, hospitalizations, and substantial geographic spread, which can quickly overwhelm existing healthcare infrastructure, leading to further amplification of the outbreak. Examining overall national emergency response capacity is essential not only to evaluate how the country will react to another measles outbreak but also to identify gaps that are applicable to other potential epidemic-prone diseases. Our multidisciplinary assessment resulted in specific, actionable recommendations for strengthening the structure and effectiveness of emergency response planning, which, if properly implemented, will have a wide-reaching effect on the reduction of illness and death during public health emergencies.

The broad health systems assessment we conducted, following the Prevent-Detect-Respond framework of the GHSA, is an example of one tactical element of a comprehensive diagonal approach to link measles elimination with immunization program and health system strengthening. Other proposed tactical elements included reaching the chronically unreached by using measles risk assessments and campaigns to identify and target underserved populations and geographies; introducing routine use of a second dose of measles vaccine to create new opportunities to receive vaccines and other child health interventions in the second year of life and beyond; and advocating for measles elimination to support institutions, policies, and practices needed to sustain high-quality immunization programs (*9*). The diagonal approach has successfully leveraged activities aimed at specific disease elimination or eradication efforts to strengthen health systems and overall immunization service delivery performance. For example, in several settings, including South Korea, Sri Lanka, the United States, and some provinces in China, school entry vaccination check laws have had a broad effect on overall coverage and equity of immunizations (*39*–*44*). Similarly, strengthening laboratory-supported surveillance systems and outbreak response capacity (including local epidemiologic capacity through FETP programs) to achieve elimination enables improved capacity to monitor surveillance performance and to detect other VPDs, such as yellow fever, Japanese encephalitis, and emerging diseases such as Ebola and Zika. For example, the existing polio eradication infrastructure in West Africa was a critical platform that was leveraged to enable rapid case detection, investigation, confirmation, and contact tracing as part of the Ebola outbreak response during 2014–2015 (*45*). In addition, established case-based surveillance systems for measles or dengue have been used to detect cases of Zika in settings where that disease is an emerging epidemic (*46*).

When measles outbreaks occur because of gaps in the confluence of multiple sectors of health systems that include immunization, IPC, surveillance, and emergency response, the GHSA framework provides useful tools to leverage outbreak investigations to strengthen the overall health system and prevent future outbreaks of measles and other infectious disease threats. In this way, GHSA investments to Prevent-Detect-Respond reduce rates of illness and death. Even relatively small measles outbreaks can have substantial cost implications (*7*); investments in measles vaccination in low- and middle-income countries yield a positive economic return on investment of 27–67 times the cost (*47*). By using the substantial multilateral investments by countries and donors to global health partnerships including GHSA, GVAP, and the Measles & Rubella Initiative to strategically strengthen health systems with a diagonal approach to measles elimination, this positive return on investment could become exponentially higher.
